# Hsa_circ_0025202 suppresses cell tumorigenesis and tamoxifen resistance via miR-197-3p/HIPK3 axis in breast cancer

**DOI:** 10.1186/s12957-021-02149-x

**Published:** 2021-02-03

**Authors:** Hongjuan Li, Qing Li, Shan He

**Affiliations:** Department of Laboratory Medicine, Jingmen No.1 People’s Hospital, Jingmen, 448000 Hubei China

**Keywords:** hsa_circ_0025202, miR-197-3p, HIPK3, Tamoxifen, Hongjuan Li and Qing Li contributed equally to this study

## Abstract

**Background:**

The involvement of circular RNAs (circRNAs) in tamoxifen (TAM) resistance has been identified. Herein, we aimed to identify the role and novel mechanisms of hsa_circ_0025202 in tamoxifen resistance in breast cancer (BC).

**Methods:**

The levels of hsa_circ_0025202, microRNA (miR)-197-3p, and homeodomain-interacting protein kinase 3 (HIPK3) were tested using quantitative real-time polymerase chain reaction and western blot. IC_50_ value of TAM, cell proliferation, cell cycle, cell invasion, migration, apoptosis, western blot, and mouse xenograft assays was used to demonstrate the effects of hsa_circ_0025202, miR-197-3p, and HIPK3 on BC cell tumorigenesis and TAM resistance. Dual-luciferase report and RNA immunoprecipitation assays were applied to explore the potential interaction between miR-197-3p and hsa_circ_0025202 or HIPK3.

**Results:**

Hsa_circ_0025202 was decreased in BC tissues and TAM resistant BC cells, and knockdown of hsa_circ_0025202 elevated the IC_50_ value of cells to TAM, led to the promotion of cell proliferation, invasion and migration, mediated cell cycle progression, and inhibited cell apoptosis in BC in vitro. Besides, the upregulation of hsa_circ_0025202 hindered tumor growth and promoted TAM sensitivity in vivo. In a mechanical study, hsa_circ_0025202 targeted miR-197-3p, and silencing of miR-197-3p reversed the regulatory effects of hsa_circ_0025202 knockdown on TAM resistance and malignant phenotypes. Additionally, HIPK3 was a target of miR-197-3p, and miR-197-3p overexpression enhanced TAM resistance and promoted cell malignant biological behaviors in BC by targeting HIPK3.

**Conclusion:**

Hsa_circ_0025202 repressed cell tumorigenesis and TAM resistance via miR-197-3p/HIPK3 axis in BC, suggesting a potential therapeutic strategy to overcome chemoresistance in BC patients.

## Background

Breast cancer (BC) is the most common malignancy affecting women and is a leading cause of cancer-related mortality worldwide [1, 2]. Tamoxifen (TAM) is the most ubiquitously applied chemotherapeutic drug for the therapy of estrogen receptor (ER) positive breast cancer, which functions as an antiestrogen by blocking the binding of estrogen to estrogen receptor s[3, 4]. However, the development of acquired drug resistance is a major obstacle in clinics, which restricts its curative effect [5, 6]. Therefore, a better understanding on the potential mechanism underlying tamoxifen resistance in BC is necessary for the development of new therapeutic approaches to overcome chemoresistance in clinical treatments of BC.

Circular RNAs (circRNAs) are a class of naturally occurring transcripts with ring structures from the splicing of exons, introns, or a combination of both, which are highly represented in the eukaryotic genome and resistance to RNase R decay [7–9]. CircRNAs often show species-, tissue-, and cell-specific expression patterns and have been implicated in multiple cellular crucial biological processes related to tumorigenesis and drug sensitivity [10–12]. Importantly, the significance of circRNAs in the resistance of BC to multiple drugs has begun to generate interest. For example, circKDM4C enhanced doxorubicin resistance and suppressed proliferation and metastasis processes in BC by upregulating PBLD through miR-548p inhibition [13]. Knockdown of circBMPR2 promoted cell tamoxifen resistance and malignant biological behaviors in BC via regulating miR-553/ubiquitin-specific protease 4 axis [14]. Thus, targeting circRNAs may be promising candidates to develop potential novel therapies for chemoresistant BC patients. Hsa_circ_0025202 is a newly identified circRNA, generated from the back-splicing of glyceraldehyde 3-phosphate dehydrogenase (GADPH) gene. Moreover, hsa_circ_0025202 have been found to inhibit BC progression and tamoxifen resistance by repressing miR-182-5p-meidated FOXO3a suppression, suggesting the anti-oncogenic role of hsa_circ_0025202 in BC [15]. However, large-scale identification of hsa_circ_0025202 in chemoresistant BC cells was not yet reported.

Herein, we aimed to probe the contributions of hsa_circ_0025202 to BC cell carcinogenesis and tamoxifen resistance and investigate other potential regulatory network underlying these effects.

## Materials and methods

### Clinical specimens

Tumor tissues and matched normal tissues were collected from 32 BC cases with a definite pathological diagnosis of BC during surgery in Jingmen No.1 People’s Hospital. All samples were immediately stored at – 80 °C until subsequent analyses. The study was approved by the Ethics Committee of Jingmen No.1 People’s Hospital and was carried out according to the guidelines of the Declaration of Helsinki. Written informed consent had been obtained from all subjects. The relevant clinical information was provided in Table 1.

**Table 1:** Association between clinicopathological variables and hsa_circ_0025202 expression in patients with breast cancer

Variable	Cases	hsa_circ_0025202		*P* value
		Low	High	
Age (years)				
≤ 50	19	8	11	0.4725
> 50	13	8	5	
Menopause				
No	17	6	11	0.1556
Yes	15	10	5	
Histologic grade				
I–II	20	5	15	0.0006^*^
III–IV	12	11	1	
Lymph node metastasis				
No	21	7	14	0.0233^*^
Yes	11	9	2	
T stage				
T1–T2	24	9	15	0.0373^*^
T3–T4	8	7	1	
*N* stage				
N0	22	7	15	0.0059^*^
N1	10	9	1	

*P < 0.05

### Cell culture

BC cell lines (T47D and MCF7), MCF-10A nonmalignant breast epithelial cells, and human embryonic kidney cell lines 293 T were bought from Jining Cell Culture Center (Shanghai, China) and grown in the Dulbecco’s modified Eagle’s medium (DMEM, HyClone, South Logan, UT, USA) with 10% fetal bovine serum (FBS) and 1% penicillin-streptomycin in a 5% CO_2_ humidified atmosphere at 37 °C.

Parental T47D and MCF7 cells at the logarithmic growth phase were digested with trypsin and then continuously exposed to increasing concentrations of TAM (Sigma, San Francisco, CA, USA) over several months to generate TAM-resistant BC cells, named MCF7/TAM and T47D/TAM. The same media supplemented with TAM (2 μM for T47D/TAM cells and 0.5 μM for MCF7/TAM cells) were employed to maintain TAM-resistant BC cells to retain their drug-resistant phenotype.

### Quantitative real-time polymerase chain reaction (qRT-PCR)

Whole-RNAs were isolated using TRIzol reagent (Sangon Biotech, Shanghai, China). Then, complementary DNA (cDNA) was generated using 1 μg of total RNA with the Prime Script RT Master Mix (Takara, Shiga, Japan), and quantitative expression of synthesized cDNA was assessed by SYBR Green PCR master mix (Takara). Subsequently, relative fold changes were determined using 2^-△△Ct^ method and normalized by U6 or GADPH. Primers for qRT-PCR were listed in Table 2.

**Table 2:** Sequences of primers for qRT-PCR

Name		Sequence
hsa_circ_0025202	Forward Reverse	5′-GACCACAGTCCATGCCATCA-3′ 5′-GTCAAAGGTGGAGGAGTGGG-3′
miR-197-3p	Forward Reverse	5′-CGGTAGTCTGATACTGTAA-3′ 5′-GTGCTCCGAAGGGGGT-3′
HIPK3	Forward Reverse	5′-ACATTGGAAGAGCATGAGGCAGAGA-3′ 5′-CTGCTGAAAAGCATCACCACAACCA-3′
GADPH	Forward Reverse	5′-GGTGAAGGTCGGAGTCAAC-3′ 5′-AGAGTTAAAAGCAGCCCTGGTG-3′
U6	Forward Reverse	5′-CTCGCTTCGGCAGCACA-3′ 5′-AACGCTTCACGAATTTGCGT-3′

### Cell transfection

The hsa_circ_0025202-specific siRNA (si-circ#1, si-circ#2, si-circ#-3), miR-197-3p mimic, miR-197-3p inhibitor, pcDNA3.1 hsa_circ_0025202/HIPK3 overexpression vector (oe-circ or oe-HIPK3), or their negative control (si-NC, mimic NC, inhibitor NC or vector) were synthesized by Invitrogen (Carlsbad, CA, USA). Then, transfection was implemented with Lipofectamine 2000 (Invitrogen).

### Drug resistance assay

The TAM resistance of cells was analyzed by measuring the half inhibitory concentration (IC_50_) value. Briefly, transfected T47D and MCF7 cells were placed into a 96-well plate at 5 × 10^3^ each well overnight and exposed to various does of TAM (0, 1, 2, 5, 10, or 15 uM) for 48 h. Afterwards, cells in each well were incubated with cell counting kit-8 (CCK-8) (Sigma) solution (10 μL/well) for 2 h at 37 °C. A microplate reader was utilized to detect absorbance values at 450 nm.

### Cell proliferation assay

Cell proliferative ability was analyzed using colony formation assay. Transfected T47D and MCF7 cells (1 × 10^3^) were seeded in a 6-well plate and grown for 2 weeks in culture medium with 10% FBS. After that, cell colonies were fixed with methanol and stained with 0.1% crystal violet for 30 min. Finally, cell colonies were photographed and counted.

### Cell cycle and apoptosis assays

To detect cell cycle, T47D and MCF7 cells were fixed in 75% ethanol overnight after transfection for 48 h, then stained with propidium iodide (PI) (BD Biosciences, San Jose, CA, USA) for 30 min in the dark. For cell apoptosis analysis, transfected T47D and MCF7 cells were resuspended in binding buffer and then stained with 10 μL of Annexin V-fluorescein isothiocyanate (FITC) and PI (BD Biosciences), away from light. Finally, a flow cytometry was used to quantify the cell cycle or cell apoptosis.

### Cell invasion and migration assays

Transwell membranes (8-μm pore size) on 24-well plates coated with Matrigel (BD Biosciences) were employed to test cell invasion. Equal numbers (2 × 10^4^) of transfected T47D and MCF7 cells with serum-free medium were added to the upper chamber, and medium containing 10% FBS was filled into the lower chamber as the chemoattractant. Following incubation for 24 h, cells remaining in the upper chamber were removed, and cells on the lower surface of the membrane were fixed with methanol and stained with crystal violet. Finally, invaded cells were imaged (× 100) and counted.

Cell migration was analyzed using wound-healing assay. In brief, transfected T47D and MCF7 cells were seeded in 6-well culture plates to grow into a monolayer, then an artificial linear wound was made by scraping using a sterile pipette tip. After washing twice with medium, cells were further grown in the medium for 24 h. Wounds were imaged with an inverted microscope at 0 and 24 h (× 40), and denuded area were examined.

### Western blot

Approximately 30 μg of extracted protein was separated on 8% sodium dodecyl sulfate polyacrylamide gel electrophoresis and transferred onto nitrocellulose membranes. After blocking with 5% nonfat milk for 1 h, the membranes were incubated with primary antibody b-cell lymphoma-2 (bcl-2) (1:3000, ab692, Abcam, Cambridge, MA, USA), ki-67 (1:5000, ab16667, Abcam), HIPK3 (1:1000, PA5-28809, Invitrogen) bcl-2-associated X (bax) (1:3000, ab32503, Abcam), and GAPDH (1:10000, ab181602, Abcam), followed by horseradish peroxidase (HRP)-conjugated secondary antibody (1:1000, ab205719). The blots were analyzed using an enhanced chemiluminescence kit (Tanon, Shanghai, China).

### In vivo assay

Female BALB/c nude mice (5 weeks old) purchased from Slake Jingda Laboratory Animal Company (Hunan, China) were randomly divided into four groups with 5 mice in each group: vector + PBS, oe-circ + PBS, vector + TAM, or oe-circ + TAM. Approximately, 1 × 10^7^ MCF7 cells stably infected with hsa_circ_0025202 overexpression vector (oe-circ) or nontarget plasmid (vector) were inoculated subcutaneously into mammary fat pads of each mouse. After a 2-week inoculation, the mice of each group were treated with PBS or TAM (5 mg/kg) by gavage every 3 days. Tumor size was recorded every 5 days. Mice were killed at day 34, and tumors were excised for weight and subsequent analyses. All animal work was approved by the Animal Ethics Committee of Jingmen No.1 People’s Hospital and performed in accordance with the guidelines of the National Animal Care and Ethics Institution.

### Dual-luciferase reporter assay

The specific sequences of hsa_circ_0025202 and HIPK3 3′-UTR harboring the complementary site of miR-197-3p were cloned into the pmirGLO dual-luciferase vector (Promega, Madison, WI, USA) to generate the corresponding wild-type (wt) or the mutated luciferase reporter constructs (hsa_circ_0025202 wt, HIPK3 3′UTR wt, or hsa_circ_0025202 mut or HIPK3 3′UTR mut), respectively. Then the constructs were introduced into 293 T cells together with mimic NC or miR-197-3p mimic for 48 h. Finally, relative luciferase activities were detected using a Dual-Luciferase reporter assay kit (Promega).

### RNA immunoprecipitation (RIP) assay

T47D and MCF7 cells were homogenized using RIP buffer, and then, cell lysate was incubated with RIPA buffer containing magnetic beads conjugated with human anti-Ago2 antibody or a negative control IgG antibody (Millipore, Billerica, MA, USA). Finally, total RNA was extracted from the beads and subjected to qRT-PCR for the quantification of hsa_circ_0025202 and miR-197-3p.

### Statistical analysis

Data analysis was handled with GraphPad Prism 7 software. Data from thrice-repeated experiments were expressed as mean ± standard deviation (SD). Statistical differences between two groups were analyzed by Student’s *t* test, and three or more groups were analyzed using one-way analysis of variance (ANOVA). The assessment of the correlation between two variables was conducted by Pearson correlation analysis. *P* < 0.05 was considered statistically significant.

## Results

### The expression profile of hsa_circ_0025202 in BC

**Fig. 1:**
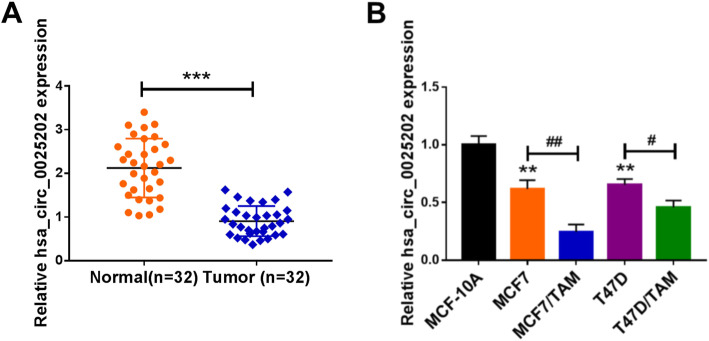
The expression profile of hsa_circ_0025202 in BC. **a** qRT-PCR analysis of hsa_circ_0025202 expression in BC tissues and matched non-tumor tissues. **b** qRT-PCR analysis of hsa_circ_0025202 expression in normal MCF-10A cells, BC cell lines (T47D and MCF7) and TAM-resistant BC cell lines (T47D/TAM and MCF7/TAM). **P <0.01, ***P < 0.001, ^#^*P* < 0.05, ^##^*P* < 0.01

Fi025202 in BC tissues was evaluated, and the results of qRT-PCR analysis showed hsa_circ_0025202 was decreased in BC tissues, compared with the non-tumor tissues (Fig. 1a). Furthermore, we analyzed the relationship between hsa_circ_0025202 expression and clinical features in 32 patients with BC. It was found that high hsa_circ_0025202 expression level is associated with histologic grade (*P* = 0.0006), higher lymph node metastasis (*P* = 0.0233), and more advanced tumor (*P* = 0.0373) and node stage (*P* = 0.0059) (Table 1). All these data suggested the potential link between hsa_circ_0025202 and BC progression. Also, hsa_circ_0025202 was lower in BC cell lines (T47D and MCF7) than that in normal MCF-10A cells, interestingly, in comparison to the parental cell lines, hsa_circ_0025202 was significantly downregulated in TAM-resistant BC cell lines (T47D/TAM and MCF7/TAM) (Fig. 1b). Thus, deregulation of hsa_circ_0025202 might play roles in cell carcinogenesis and TAM resistance in BC.

### Effects of hsa_circ_0025202 on cell TAM sensitivity and carcinogenesis in BC

**Fig. 2:**
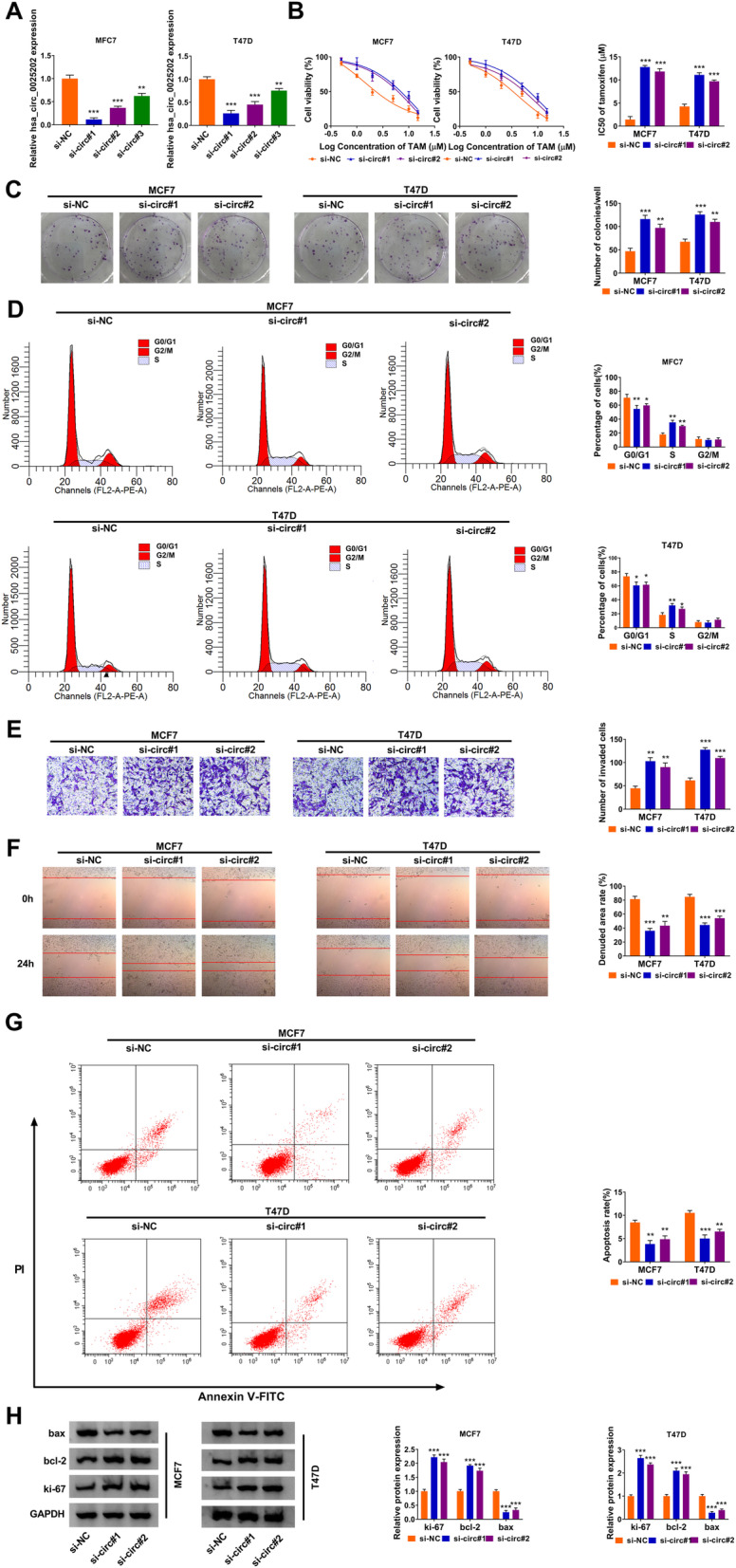
Effects of hsa_circ_0025202 on cell TAM sensitivity and carcinogenesis in BC. **a** qRT-PCR analysis of hsa_circ_0025202 expression in T47D and MCF7 cells transfected with three hsa_circ_0025202 specific siRNAs (si-circ#1, si-circ#2, si-circ#3). **b** The IC_50_ value of TAM by CCK-8 assay, **c** cell proliferation by colony formation assay, **d** cell cycle by flow cytometry assay, **e** cell invasion by transwell assay, **f** cell migration by wound healing assay, **g** cell apoptosis by flow cytometry assay, **h** the levels analysis of bax, ki-67 and bcl-2 by western blot, in T47D and MCF7 cells transfected with si-NC, si-circ#1, or si-circ#2. *P < 0.05, **P < 0.01, ***P < 0.001

Next, the detailed functions of hsa_circ_0025202 on BC cell chemosensitivity and carcinogenesis were investigated. Three siRNAs targeting hsa_circ_0025202 (si-circ#1, si-circ#2, si-circ#3) were transfected into T47D and MCF7 cells, and as expected, hsa_circ_0025202 expression was prominently downregulated (Fig. 2a). Afterwards, CCK-8 assay showed the IC_50_ value to TAM in hsa_circ_0025202-decreased T47D and MCF7 cells was significantly elevated compared with respective control cells (Fig. 2b). Meanwhile, results of colony formation, flow cytometry, wound healing, and transwell assays exhibited that knockdown of hsa_circ_0025202 promoted cell colony formation ability (Fig. 2c), induced cell cycle progression (Fig. 2d), led to a significant promotion in cell invasion and migration (Fig. 2e, f), and suppressed the apoptosis (Fig. 2g) in parental T47D and MCF7 cells. Besides, western blot analysis also suggested ki-67 and bcl-2 expression were elevated while bax expression was decreased in hsa_circ_0025202-decreased T47D and MCF7 cells (Fig. 2h), further revealing hsa_circ_0025202 downregulation promoted cell proliferation but repressed cell apoptosis. Taken together, knockdown of hsa_circ_0025202 enhanced cell TAM resistance and promoted cell malignant biological behaviors in BC.

### Effects of hsa_circ_0025202 on tumor growth and TAM resistance in vivo in BC

**Fig. 3:**
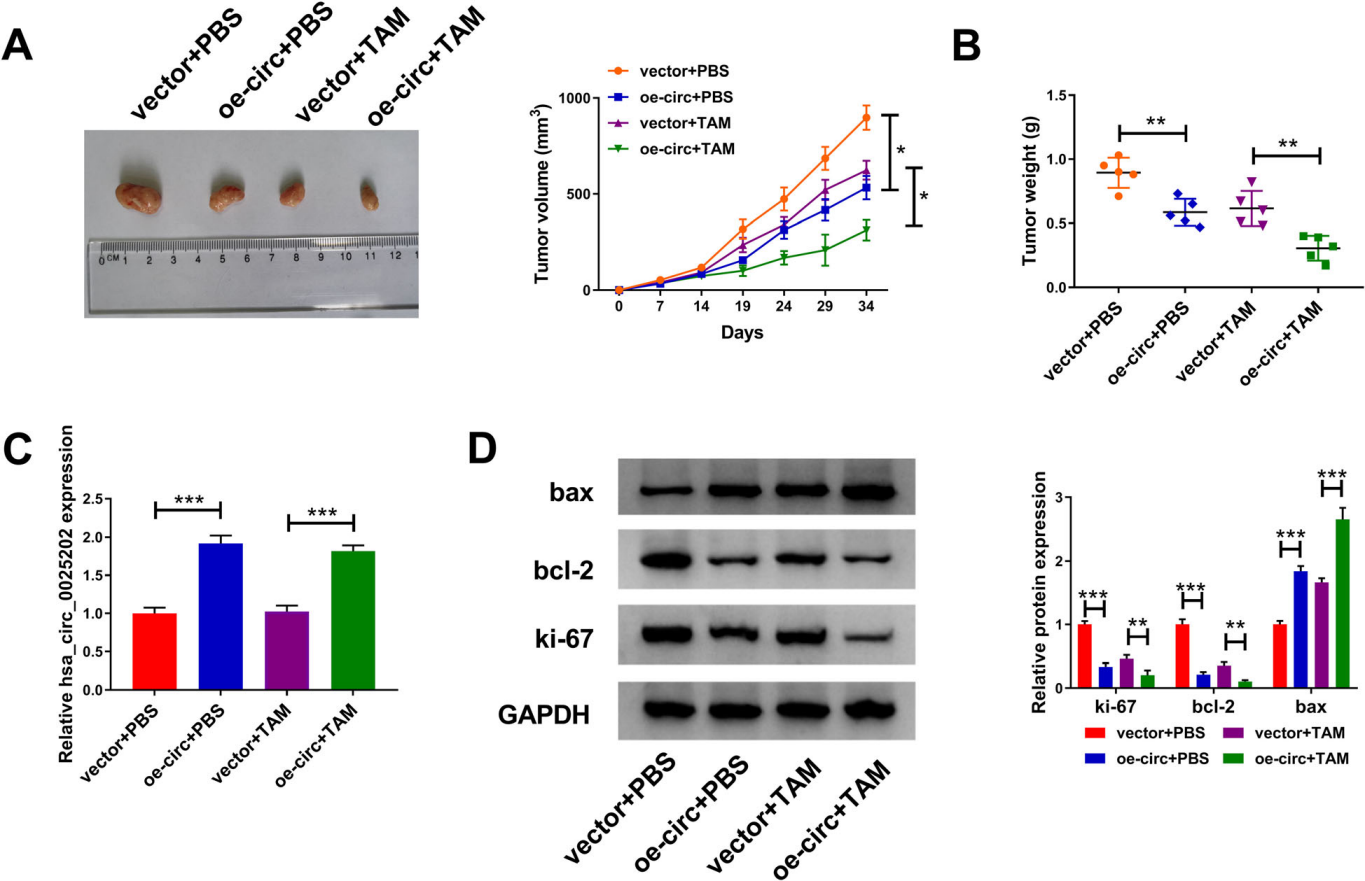
Effects of hsa_circ_0025202 on tumor growth and TAM resistance in vivo in BC. **a** Tumor volume was calculated every 5 days. **b** The average weights of dissected tumors were detected in each group. **c** qRT-PCR analysis of hsa_circ_0025202 level in dissected tumors. **d** Protein level detection of bax, ki-67 and bcl-2 in dissected tumors using western blot. *P < 0.05, **P < 0.01, ***P < 0.001

The impacts of hsa_circ_0025202 on tumor growth and TAM resistance in vivo were then investigated using xenograft models. As shown in Fig. 3a, b, we observed that TAM treatment or hsa_circ_0025202 overexpression dramatically suppressed tumor volume and weight relative to the control group; importantly, a more distinct reduction on tumor growth was discovered by simultaneous hsa_circ_0025202 upregulation together with TAM treatment. Additionally, subsequent molecular analysis exhibited that hsa_circ_0025202 expression was elevated in tumors derived from hsa_circ_0025202-transfected MCF7 cells with or without TAM treatment (Fig. 3c). Furthermore, results from western blot analysis displayed that levels of ki-67 and bcl-2 were decreased, while bax expression was increased by hsa_circ_0025202 upregulation or TAM treatment, especially by simultaneous hsa_circ_0025202 restoration plus TAM treatment (Fig. 3d). In all, the upregulation of hsa_circ_0025202 retarded BC tumor growth and enhanced TAM sensitivity in vivo.

### MiR-197-3p is a target of hsa_circ_0025202

**Fig. 4:**
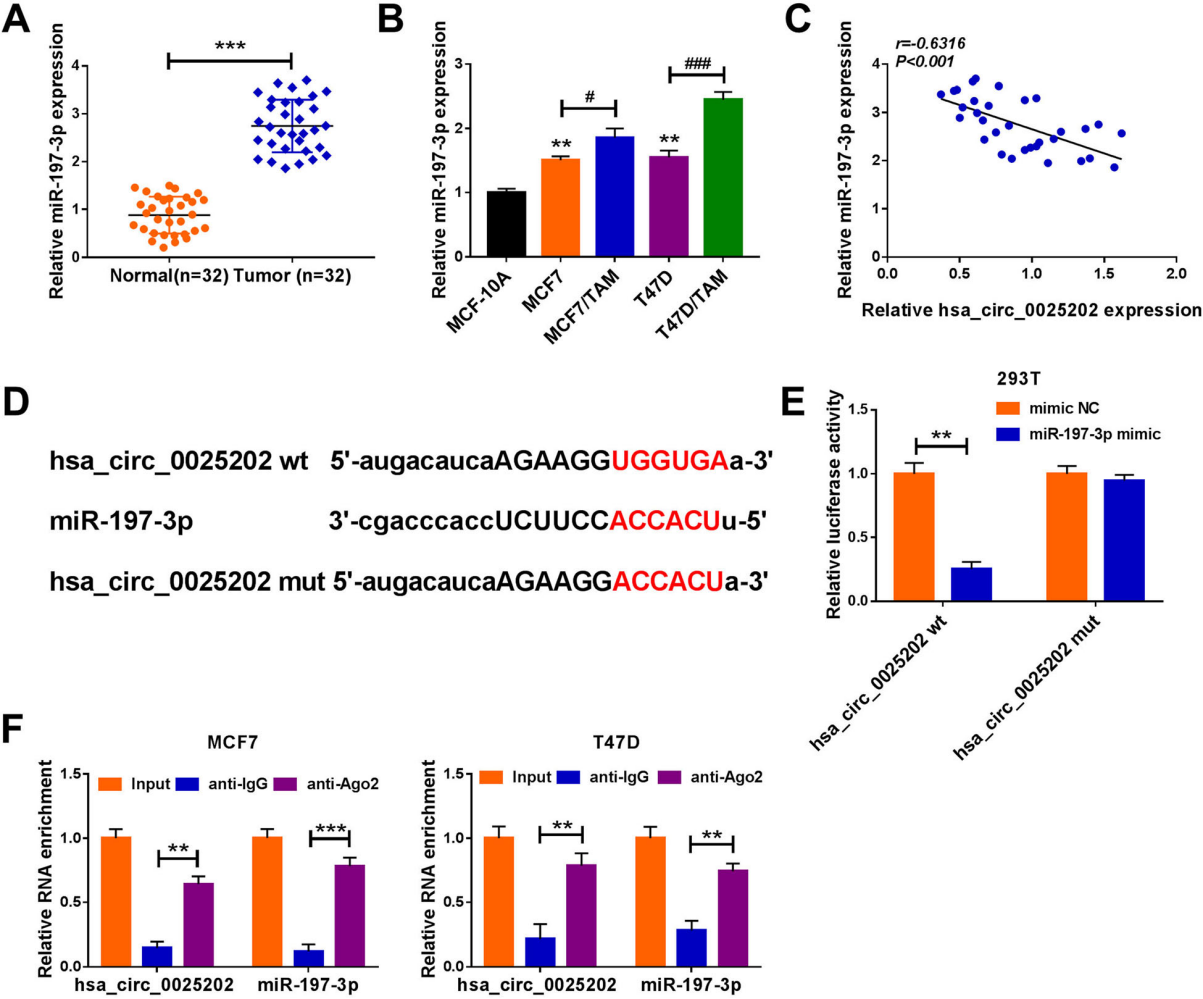
MiR-197-3p is a target of hsa_circ_0025202. **a** qRT-PCR analysis of miR-197-3p expression in BC tissues and matched non-tumor tissues. **b** qRT-PCR analysis of miR-197-3p expression in normal MCF-10A cells, BC cell lines (T47D and MCF7) and TAM-resistant BC cell lines (T47D/TAM and MCF7/TAM). **c** The correlation between hsa_circ_0025202 level and miR-197-3p expression in BC tissues using the Pearson correlation analysis. **d** Sequence alignment of miR-197-3p with the putative binding sites in the wild-type regions of hsa_circ_0025202. **e** Luciferase activities detection in 293 T cells co-transfected with the reporter plasmids and indicated miRNAs using the dual-luciferase reporter assay. **f** qRT-PCR analysis of hsa_circ_0025202 and miR-197-3p levels in T47D and MCF7 cells after RIP assay. **P < 0.01, ***P < 0.001, ^#^*P* < 0.05, ^###^*P* < 0.001

In BC tissues, we also found miR-197-3p was upregulated compared with the non-tumor tissues (Fig. 4a); besides, its expression was higher in T47D and MCF7 cells, in particular in TAM-resistant BC cell lines (T47D/TAM and MCF7/TAM), than that in normal MCF-10A cells (Fig. 4b). Moreover, it was observed that hsa_circ_0025202 expression was negatively correlated with miR-197-3p in BC tissues (Fig. 4c). Thus, we speculated that hsa_circ_0025202-mediated regulatory functions might operate through miR-197-3p. After searching the online databases Starbase3.0, miR-197-3p was identified to have putative binding sites of hsa_circ_0025202 and might be a potential underlying microRNA (miRNA) that could be interacted with hsa_circ_0025202 (Fig. 4d). Then dual-luciferase reporter assay was implemented, and the results showed the luciferase activity of wild-type reporter was significantly suppressed by miR-197-3p upregulation, while no change was observed in mutated reporter after miR-197-3p overexpression in 293 T cells (Fig. 4e). Moreover, RIP assay showed that hsa_circ_0025202 and miR-197-3p could be enriched by anti-Ago2 antibody relative to anti-IgG antibody in T47D and MCF7 cells (Fig. 4f). All these data verified that hsa_circ_0025202 directly bound to miR-197-3p in BC cells.

### Hsa_circ_0025202 knockdown promotes cell TAM resistance and carcinogenesis in BC via miR-197-3p

**Fig. 5:**
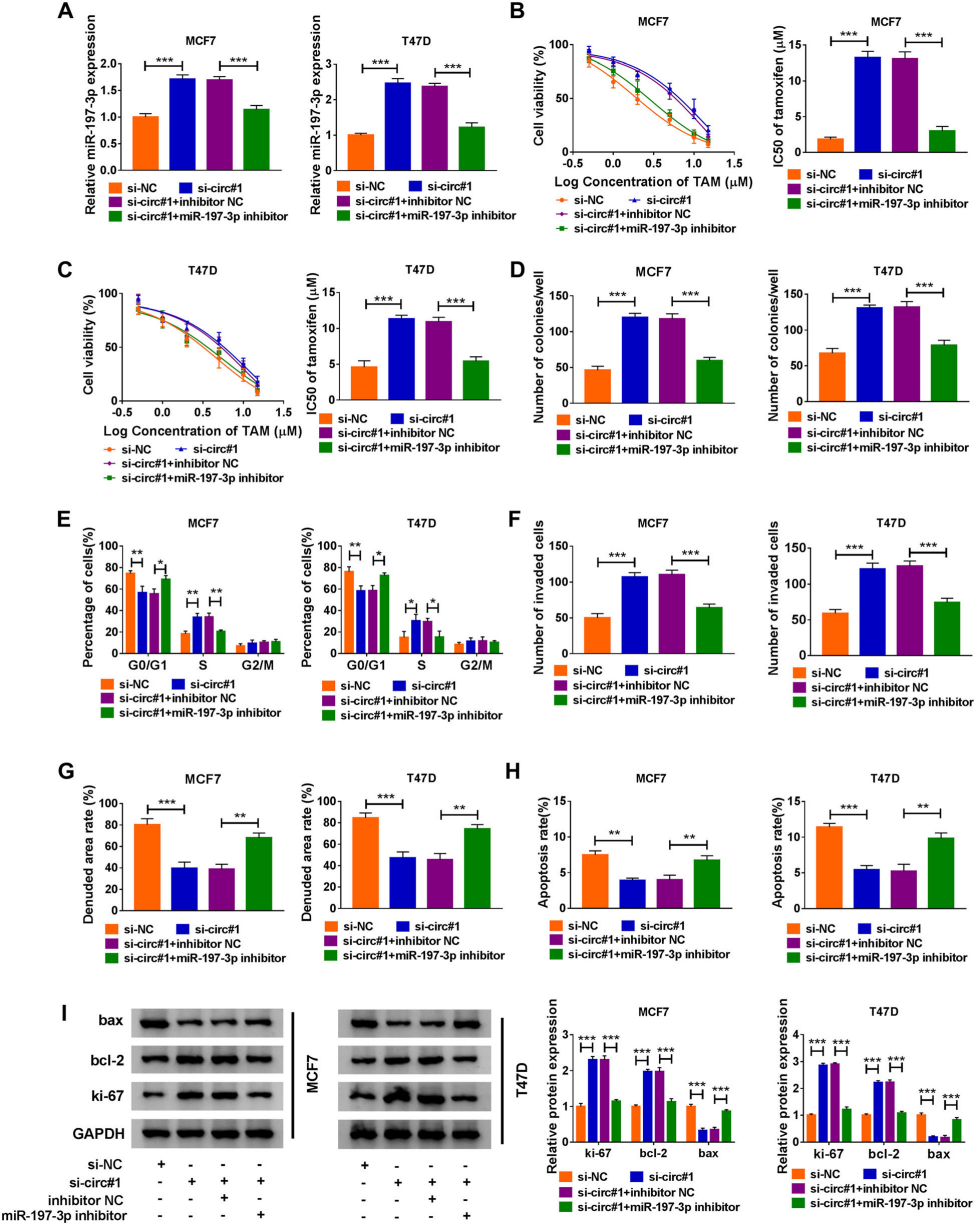
Hsa_circ_0025202 knockdown promotes cell TAM resistance and carcinogenesis in BC via miR-197-3p. **a** MiR-197-3p level by qRT-PCR; **b**, **c** the IC_50_ value of TAM by CCK-8 assay; **d** cell proliferation by colony formation assay; **e** cell cycle by flow cytometry assay; **f** cell invasion by transwell assay; **g** cell migration by wound healing assay; **h** cell apoptosis by flow cytometry; **i** protein levels of bax, ki-67, and bcl-2 by western blot, in T47D and MCF7 cells transfected with si-NC, si-circ#1, si-circ#1 + inhibitor NC, and OR si-circ#1 + miR-197-3p inhibitor. *P < 0.05, **P < 0.01, ***P < 0.001

Given the direct interaction between hsa_circ_0025202 and miR-197-3p, whether the regulatory effects of hsa_circ_0025202 on BC cell TAM sensitivity and carcinogenesis were mediated by miR-197-3p was investigated. T47D and MCF7 cells were transfected with si-NC, si-circ#1, si-circ#1 + inhibitor NC, OR si-circ#1 + miR-197-3p inhibitor, then qRT-PCR analysis suggested the introduction of miR-197-3p inhibitor reversed hsa_circ_0025202 knockdown-induced elevation of miR-197-3p expression in cells (Fig. 5a), suggesting hsa_circ_0025202 negatively regulated miR-197-3p expression in BC cells. Then, the rescue assay was performed, and the results exhibited the regulatory effects of si-hsa_circ_0025202 on T47D and MCF7 cell TAM sensitivity, proliferation, cell cycle, invasion, migration, and apoptosis were markedly abrogated by miR-197-3p downregulation relative to the corresponding counterparts (Fig. 5b–h). Furthermore, the elevation of ki-67 and bcl-2 expression and decrease of bax expression in hsa_circ_0025202-decreased T47D and MCF7 cells were also reversed by the introduction of miR-197-3p inhibitor (Fig. 5i). Altogether, silencing of hsa_circ_0025202 enhanced cell TAM resistance and promoted tumorigenesis in BC by binding to miR-197-3p.

### HIPK3 is a target of miR-197-3p

**Fig. 6:**
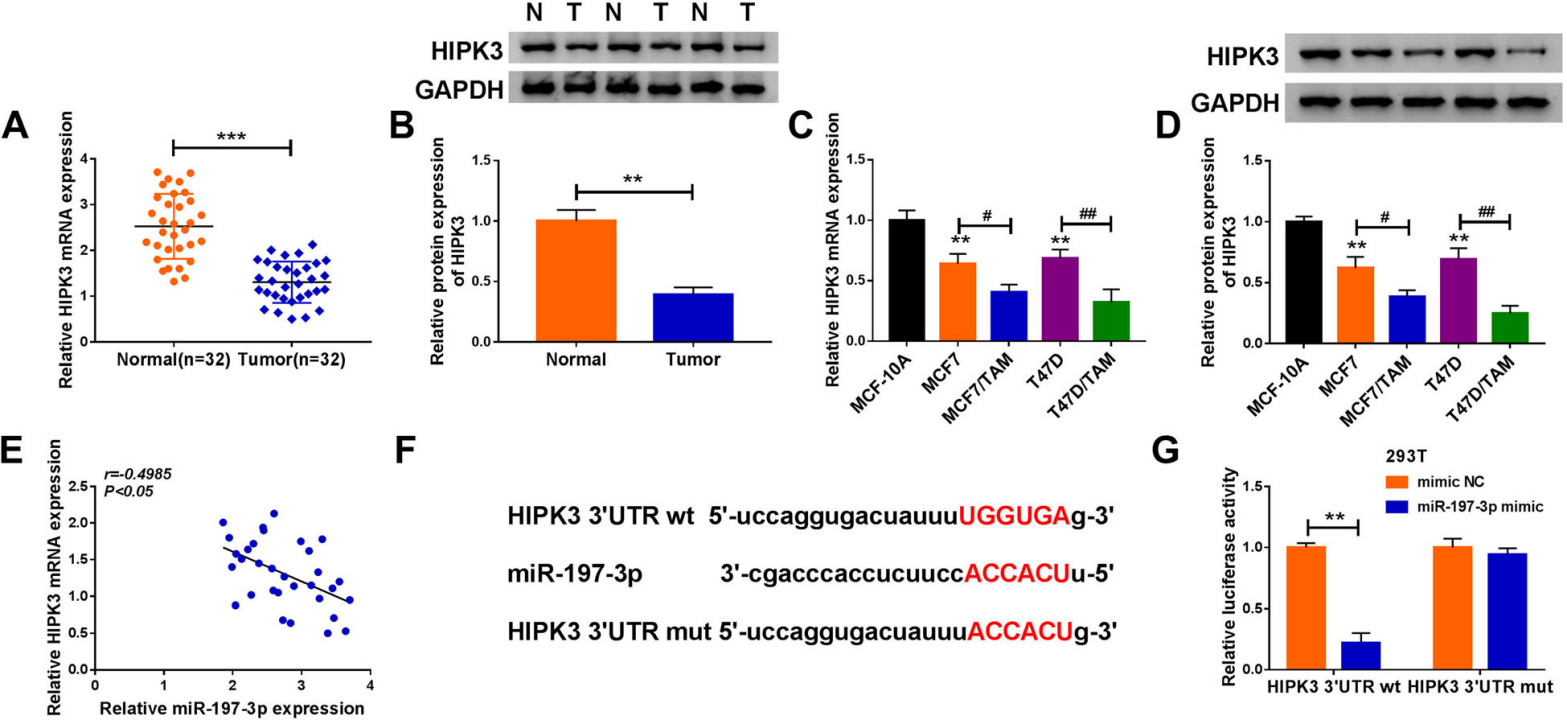
HIPK3 is a target of miR-197-3p. **a**, **b** qRT-PCR and western blot analysis of HIPK3 expression in BC tissues and matched non-tumor tissues. **c**, **d** qRT-PCR and western blot analysis of HIPK3 expression in normal MCF-10A cells, BC cell lines (T47D and MCF7), and TAM-resistant BC cell lines (T47D/TAM and MCF7/TAM). **e** The correlation between HIPK3 level and miR-197-3p expression in BC tissues using the Pearson correlation analysis. **f** The potential binding sites of HIPK3 and miR-197-3p. **g** Luciferase activities detection in 293 T cells co-transfected with the reporter plasmids and indicated miRNAs using the dual-luciferase reporter assay. **P < 0.01, ***P < 0.001, ^#^*P* < 0.05, ^##^*P* < 0.01

MiRNAs often mediate their roles via regulating target genes. We detected that HIPK3 was decreased in BC tissues (Fig. 6a, b); similarly, its expression was also downregulated in T47D and MCF7 cells, especially in TAM-resistant BC cell lines (T47D/TAM and MCF7/TAM) (Fig. 6c, d). Importantly, a negative correlation between miR-197-3p and HIPK3 expression was discovered in BC tissues (Fig. 6e). Thus, the link between miR-197-3p and HIPK3 was investigated. According to the prediction of online software Starbase3.0, the predicted miR-197-3p-binding sites were investigated in the 3′ UTR of HIPK3 (Fig. 6f). Afterwards, the reduction of luciferase activity in 293 T cells co-transfected with HIPK3 3′ UTR wt and miR-197-3p mimic confirmed the direct interaction between miR-197-3p and HIPK3 (Fig. 6g). Therefore, we validated that HIPK3 was a target of miR-197-3p.

### MiR-197-3p promotes cell TAM resistance and carcinogenesis in BC via HIPK3

**Fig. 7:**
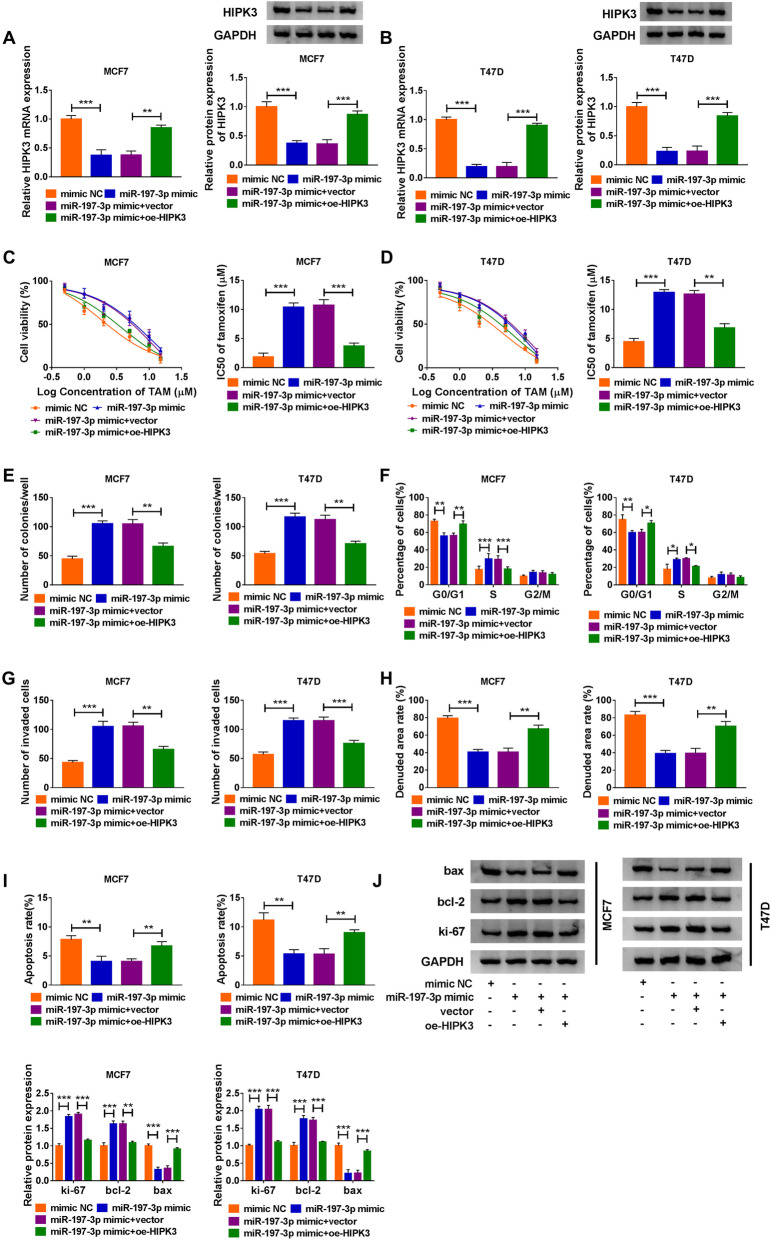
MiR-197-3p promotes cell TAM resistance and carcinogenesis in BC via HIPK3. **a**, **b** HIPK3 level by qRT-PCR and western blot; **c**, **d** the IC_50_ value of TAM by CCK-8 assay; **e** cell proliferation by colony formation assay; **f** cell cycle by flow cytometry assay; **g** cell invasion by transwell assay; **h** cell migration by wound healing assay; **i** cell apoptosis by flow cytometry; **j** protein levels of bax, ki-67, and bcl-2 by western blot, in T47D and MCF7 cells transfected with mimic NC, miR-197-3p mimic, miR-197-3p mimic + vector, or miR-197-3p mimic + oe-HIPK3. *P < 0.05, **P < 0.01, ***P < 0.001

The link between miR-197-3p and HIPK3 in BC cell TAM resistance and carcinogenesis was then studied. T47D and MCF7 cells were transfected with mimic NC, miR-197-3p mimic, miR-197-3p mimic + vector, or miR-197-3p mimic + oe-HIPK3, and after transfection, we found miR-197-3p overexpression reduced HIPK3 expression in cells, while this condition was abolished by HIPK3 upregulation (Fig. 7a, b), indicating miR-197-3p inversely modulated HIPK3 expression. After that, results of functional experiments showed miR-197-3p overexpression elevated the IC_50_ value of cells to TAM (Fig. 7c, d), promoted cell colony formation (Fig. 7e), mediated cell cycle progression (Fig. 7Ff), induced cell invasion and migration promotion (Fig. 7g, h), inhibited cell apoptosis (Fig. 7i), and caused an elevation of ki-67 and bcl-2 expression and decrease of bax expression in T47D and MCF7 cells (Fig. 7j); however, these effects were attenuated by the restoration of HIPK3 (Fig. 7c–j). Collectively, miR-197-3p promoted cell TAM resistance and carcinogenesis in BC via HIPK3.

## Discussion

BC is the most common female malignancy and approximately 70% of breast tumors express ER alpha (ERα) [16]. Tamoxifen is widely applied as the standard endocrine therapy since its discovery in 1970 for the treatment of women with ERα-positive BC, especially in premenopausal patients [17, 18]. However, the development of tamoxifen resistance in BC has emerged, which limits the therapeutic efficacy of tamoxifen in clinics [6]. The potential mechanisms underlying tamoxifen resistance involves multiple factors. Among them, the implication of noncoding RNAs, including circRNAs and miRNAs, in tamoxifen resistance has been identified [13, 19, 20], which are potential targets for the development of molecule-based therapeutic approaches.

In this study, hsa_circ_0025202 was found to be downregulated in BC tissues, besides, it was also demonstrated to be decreased in BC cells, particularly in TAM-resistant BC cells, suggesting deregulation of hsa_circ_0025202 might be associated with cell carcinogenesis and TAM resistance in BC. Afterwards, loss-of-function experiments were conducted, results showed hsa_circ_0025202 silencing in BC cells elevated the IC_50_ value of cells to TAM, resulted in a significant promotion in cell proliferation, cell cycle, cell invasion and migration, and repressed apoptosis in vitro. Importantly, xenograft assays exhibited that the upregulation of hsa_circ_0025202 hindered BC tumor growth and enhanced the cytotoxicity of TAM in tumor in vivo. Therefore, hsa_circ_0025202 plays a protective role in cell TAM resistance and tumorigenesis in BC.

Emerging evidence has demonstrated that alterations of miRNA expression and function are also associated with tamoxifen resistance. For instance, several oncogenic miRNAs, such as miR-221/222 and miR-519a, confer tamoxifen resistance [21, 22], whereas re-expression of miR-148a, miR-152, or miR-449a reduce tamoxifen resistance through their respective mRNA target genes [23, 24]. MiR-197-3p is a functional miRNA and has been identified to act as an oncogene to promote BC progression [25, 26]. However, the role of miR-197-3p in the tamoxifen resistance of BC has not been investigated. In the present study, we observed miR-197-3p was significantly upregulated in BC tissues and cells, especially in TAM-resistant BC cells. Subsequent experiments showed miR-197-3p overexpression enhanced cell TAM resistance and promoted cell oncogenic phenotypes in BC.

Previous reports have revealed circRNAs can function as miRNA sponges, thus repressing their ability to target mRNAs [27, 28]. Therefore, we speculated hsa_circ_0025202 might perform regulatory effects by a sponge mechanism. In a mechanical study, we confirmed that miR-197-3p directly bound to hsa_circ_0025202 or HIPK3. Additionally, rescue experiments suggested that miR-197-3p inhibition abolished the regulatory effects of hsa_circ_0025202 knockdown on BC cells, and miR-197-3p performed oncogenic roles by targeting HIPK3.

## Conclusion

In summary, this study demonstrated that hsa_circ_0025202 suppressed carcinogenesis and reduced tamoxifen resistance in BC via regulating miR-197-3p/HIPK3 axis, providing an actionable therapeutic strategy in BC patients with chemoresistance.

## Data Availability

The data sets used and/or analyzed during the current study are available from the corresponding author on reasonable request.
